# MSMB gene rs10993994 polymorphism increases the risk of prostate cancer

**DOI:** 10.18632/oncotarget.15312

**Published:** 2017-02-14

**Authors:** Tao Peng, Lifeng Zhang, Lijie Zhu, Yuan-Yuan Mi

**Affiliations:** ^1^ Department of Urology, The Third Affiliated Hospital of Nantong University, Wuxi, PR China; ^2^ Department of Urology, Changzhou No.2 People′s Hospital Affiliated to Nanjing Medical University, Changzhou, Jiangsu Province, China

**Keywords:** MSMB, prostate cancer, rs10993994, meta-analysis, single nucleotide polymorphism

## Abstract

Genome-wide association studies (GWASs) identified microseminoprotein-β (MSMB) gene rs10993994 polymorphism was significantly associated with prostate cancer (PC) risk. However, the association between MSMB gene rs10993994 polymorphism and PC risk remains controversial. Therefore, we performed a systematic review and meta-analysis by searching in the databases of PubMed, and Embase. Pooled odds ratios (ORs) and 95% confidence intervals (CIs) were calculated by using fixed-effect or random-effect models. A total of 11 publications containing 13 case-control studies for rs10993994 polymorphism were included in our analysis. Our data indicated that MSMB gene rs10993994 polymorphism was associated with an increased risk of PC. Stratification analyses of ethnicity suggested rs10993994 polymorphism increased the risk of PC among Caucasians, but not among Asians. In conclusion, this meta-analysis indicates that MSMB gene rs10993994 polymorphism increases the risk of PC.

## INTRODUCTION

Prostate cancer (PC) is the second most common cancer among men worldwide [1]. Several risk factors including age, family history and ethnic origin have been identified [2]. The etiology of PC remains largely unknown. Data suggested that 30 - 40% of all early-onset PC (< 55 years) are caused by inherited factors [3], indicating the genetic factor of this disease. Hereditary susceptibility is recognized as the strongest risk factor for PC [4].

Three most abundant proteins secreted by the prostate include Microseminoprotein-β (MSMB), prostatic acid phosphatase (PAP) and prostate-specific antigen (PSA) [5]. This MSMB gene codes for protein of 94 amino acids (PSP94), a predominant protein secreted by the prostate tissue and an important candidate gene for PC. More than 40 PC susceptibility loci have been identified [6], which could explain about 25% of the familial risk in this disorder. Among these single nucleotide polymorphisms (SNPs), several genome-wide association studies (GWASs) identified rs10993994 polymorphism in the promoter region of MSMB gene [7, 8], which was significantly associated with PC susceptibility. Subsequent studies [9–18] also investigated the association between MSMB gene rs10993994 polymorphism and PC susceptibility, but with conflicting conclusions. These studies were conflicting and inconclusive probably due to different ethnic populations, clinical heterogeneity, and small sample sizes. Therefore, we conducted a comprehensive meta-analysis to explore the possible association between MSMB gene rs10993994 polymorphism and PC risk.

## RESULTS

### Characteristics of the included studies

**Figure 1:**
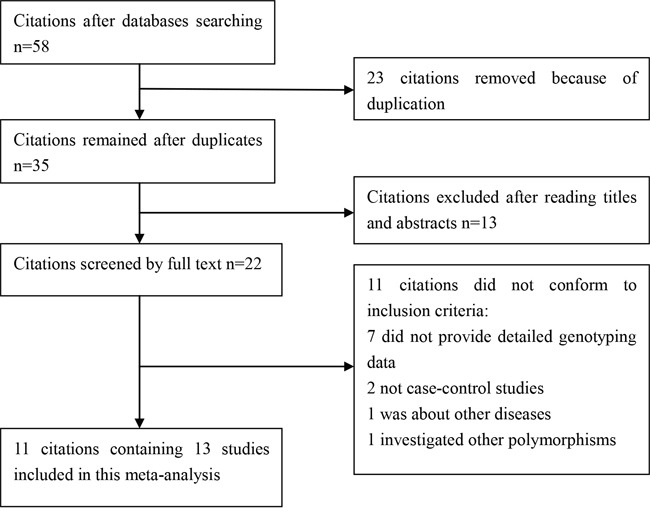
Selection for eligible citations included in this meta-analysis

We yielded 58 citations after database searching. 35 citations were removed after removing duplicates and screening the titles and abstracts. 22 citations were selected for further full text review. 11 citations were excluded: 7 did not provide detailed genotyping data; 2 not case-control studies; 1 was about other diseases; 1 investigated other polymorphisms. We finally identified 11 eligible citations [8–18] including 13 studies (31,584 cases and 30,251 controls) in this meta-analysis. Selection for eligible studies included in this meta-analysis was presented in Figure 1. The characteristics of included studies are summarized in Table 1. The Newcastle-Ottawa Scale (NOS) scores of all included studies ranged from 5 to 7 stars, suggesting that these studies were of high methodological quality. All included studies conformed to Hardy–Weinberg equilibrium (HWE).

**Table 1:** Characteristics of included studies

Author and year	SOC	Genotype methods	Ethnicity	Case			Control			HWE	NOS
				CC	CT	TT	CC	CT	TT		
Sjoblom2016	HB	PCR	Caucasian	154	160	54	394	396	111	Y	6
Mhatre2015	PB	PCR	Asian	9	24	17	5	10	15	Y	7
Shui2014	PB	TaqMan	Caucasian	3289	5168	2030	4102	5245	1677	Y	6
Stott-Miller2013	PB	Taqman	Caucasian	377	621	241	465	599	168	Y	7
FitzGerald2013	PB	Taqman	Caucasian	382	633	242	472	608	173	Y	7
Haiman2013	PB	AutoDELFIA	Mixed	314	588	319	359	585	286	Y	6
Ho2012	PB	PCR	Caucasian	83	94	65	102	119	43	Y	6
Chang2011	HB	PCR	African-American	1553	1904	583	1349	1799	600	Y	7
Xu2010	PB	Taqman	Asian	57	122	72	71	140	47	Y	6
Chang2009a	PB	PCR	Caucasian	963	1354	546	627	810	264	Y	6
Chang2009b	HB	PCR	Caucasian	1380	2129	935	1275	1584	491	Y	6
Eeles2008a	HB	HapMap	Caucasian	543	921	390	815	854	225	Y	6
Eeles2008b	HB	HapMap	Caucasian	960	1622	686	1204	1618	544	Y	6

SOC, source of controls; PB, population-based controls; HB, hospital-based controls; HWE, Hardy–Weinberg equilibrium; NOS, Newcastle-Ottawa Scale.

### Quantitative synthesis

**Figure 2:**
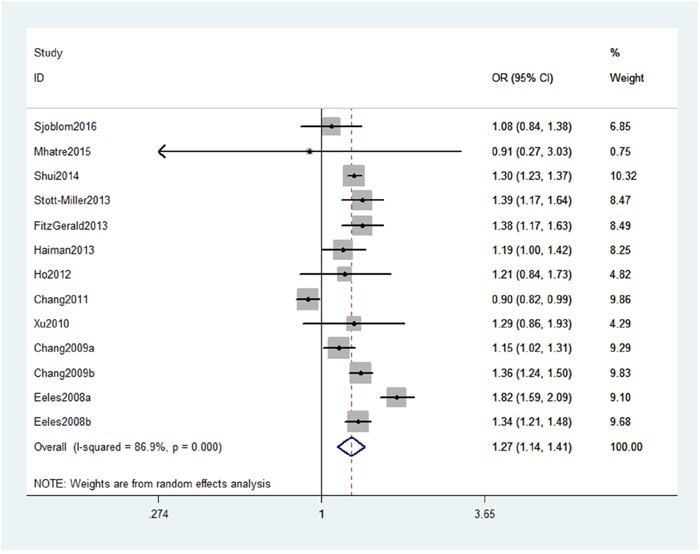
Forest plot shows odds ratio for the associations between rs10993994 polymorphism and PC risk (CT+TT vs. CC)

**Figure 3:**
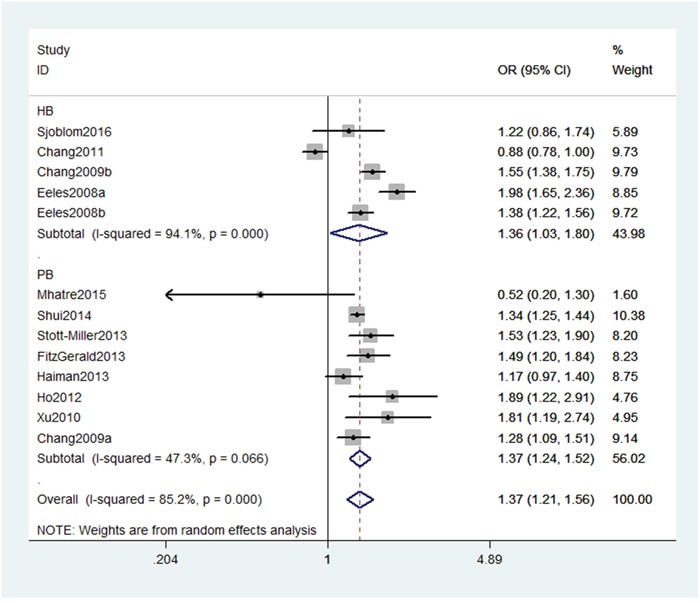
Stratification analyses by source of controls between rs10993994 polymorphism and PC risk (TT vs. CT+CC)

As shown in Table 2, we detected a significant association between MSMB gene rs10993994 polymorphism with an increased PC risk (CT+TT vs. CC: OR, 1.27; 95% CI, 1.14–1.41, *P* < 0.001, Figure 2). Stratification analyses were conducted according to ethnicity and source of controls (SOCs). Our data indicated that rs10993994 polymorphism was significantly associated with an increased risk of PC among Caucasian populations, but not among Asian populations (Table 3). As for other populations, a weak association was detected among African-Americans and mixed populations. Regarding stratification analysis by source of controls (SOCs), similar result was obtained in both population-based controls and hospital-based controls (TT vs. CT+CC, Figure 3).

**Table 2:** Meta-analysis of association between MSMB rs10993994 polymorphism and prostate cancer risk

Comparison	OR(95%CI)	*P*-value	*P* for heterogeneity	I^2^ (%)	Model
T vs. C	1.23(1.13,1.34)	<0.001	<0.001	90.6	Random
CT+TT vs. CC	1.27(1.14,1.41)	<0.001	<0.001	86.9	Random
TT vs. CT+CC	1.37(1.21,1.56)	<0.001	<0.001	85.2	Random

**Table 3:** Summary of the subgroup analyses in this meta-analysis

Comparison	Category	Category	Studies	OR (95% CI)	*P*-value
T vs. C	Ethnicity	Caucasian	9	1.29(1.21,1.37)	<0.001
		Asian	2	1.04(0.55,1.99)	0.899
		African–American	1	1.14(1.02,1.27)	0.026
		Mixed	1	0.92(0.86,0.98)	0.010
	SOC	HB	5	1.22(1.01,1.48)	0.043
		PB	8	1.23(1.17,1.29)	<0.001
CT+TT vs. CC	Ethnicity	Caucasian	9	1.34(1.24,1.46)	<0.001
		Asian	2	1.25(0.85,1.83)	0.256
		African–American	1	1.19(1.00,1.42)	0.054
		Mixed	1	0.90(0.82,0.99)	0.026
	SOC	HB	5	1.27(1.00,1.61)	0.054
		PB	8	1.28(1.22,1.34)	<0.001
TT vs. CT+CC	Ethnicity	Caucasian	9	1.48(1.34,1.62)	<0.001
		Asian	2	1.03(0.31,3.51)	0.956
		African–American	1	1.17(0.97,1.40)	0.099
		Mixed	1	0.88(0.78,1.00)	0.053
	SOC	HB	5	1.36(1.03,1.80)	0.032
		PB	8	1.37(1.24,1.52)	<0.001

SOC, source of controls; PB, population-based controls; HB, hospital-based controls.

**Figure 4:**
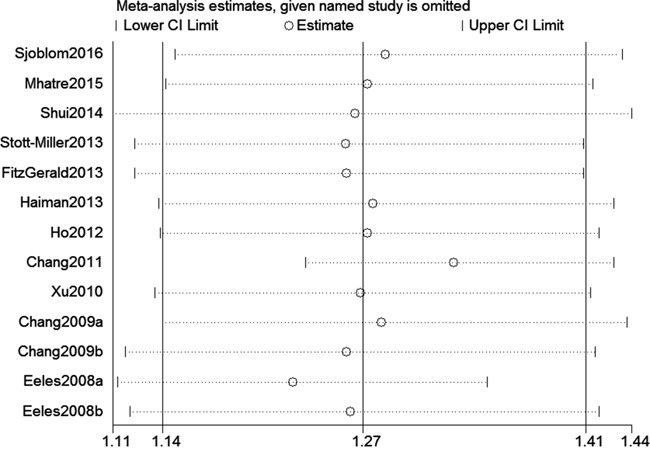
Sensitivity analysis about rs10993994 polymorphism and PC risk (TT vs. CT+CC)

**Figure 5:**
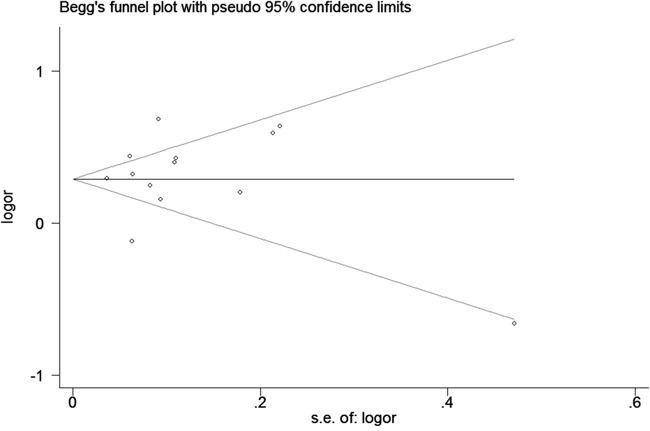
Begg's tests about rs10993994 polymorphism and PC risk (CT+TT vs. CC)

We assessed sensitivity by omitting each study once at a time in every genetic model for rs10993994 polymorphism. The pooled ORs for the effects about this polymorphism indicated that our data were stable and trustworthy (TT vs. CT+CC, Figure 4). Both Egger's and Begg's tests (CT+TT vs. CC, Figure 5) were used to evaluate the publication bias of this meta-analysis. Our data revealed that there was no obvious publication bias for rs10993994 polymorphism. Due to significant between-study heterogeneity among every genetic model, we conductedmeta-regression to explore whether ethnicity and source of controlswere the resource of heterogeneity. However, our data suggested that ethnicity and source of controls did not seem to be responsible for the heterogeneity (data not shown).

## DISCUSSION

This is the first meta-analysis to investigate the association between MSMB gene rs10993994 polymorphism and PC susceptibility. Our data indicated that MSMB gene rs10993994 polymorphism increased the risk of PC. Stratification analyses of ethnicity suggested that rs10993994 polymorphism was associated with an increased risk of PC only among Caucasians.

The MSMB gene is located on chromosome 10q11.2 [19]. MSMB codes for a secreted seminoprotein. This protein has tumour suppressor properties and is silent in prostate tumour tissues [20]. Over expression of MSMB could induce PC cell apoptosis and suppress prostate cancer growth, invasion and metastasis [11, 21]. Harries et al. indicated that alterations in MSMB gene expression are associated with the development of PC [4]. The SNP rs10993994 is located in the promoter region of MSMB gene. Studies [22, 23] have demonstrated that the risk allele of rs10993994 polymorphism was significantly associated with decreased expression of MSMB mRNA and protein in prostate tissues. It is reasonable to hypothesize that MSMB gene rs10993994 polymorphism plays a pivotal role in the pathogenesis of PC.

To date, many studies [8–18] explored the association between MSMB gene 10993994 polymorphism and PC risk. However, these studies detected conflicting results. To achieve reliable conclusions, we conducted a meta-analysis to demonstrate the associations between this SNP and PC susceptibility. Our data indicated that MSMB rs10993994 polymorphism increased the risk of PC. Stratification analyses of ethnicity in this study suggested that this SNP was associated with an increased risk of PC among Caucasians, while no association was detected among Asians. As for other populations, we found a weak association among African-Americans and mixed populations. Obviously, diversity inheritance of different ethnicities was presented in this meta-analysis. The reasons why their conclusions in diverse ethnicities vary are still unclear. It may be partially explained by different ethnic groups with various genetic backgrounds, small sample sizes, and clinical heterogeneity. It is noteworthy that the sample sizes of Asians and African-Americans are limited. Therefore, larger studies are needed to identify the possible association in those ethnicities. Additionally, we conducted stratified analysis by source of controls and similar positive results were obtained in both population-based studies and hospital-based studies.

To seek the sources of high heterogeneity in this study, we conducted meta-regression analysis, stratification analyses, and sensitivity analysis. Meta-regression analysis of ethnicity and source of controls was conducted. Our data confirmed that ethnicity and source of controls were not the sources of heterogeneity. Stratification analysis and sensitivity analysis also did not find the sources of heterogeneity. Clinical heterogeneity and different environments might be the reasons for high heterogeneity, which needs further studies to validate.

We believe this meta-analysis has some strength. First, we identified 13 studies with large sample sizes including 31,584 cases and 30,251 controls. Second, sensitivity analysis indicated that our data regarding rs10993994 polymorphism were stable and dependable (Figure 4). Third, this study had a power of 99.9% to detect the effect of rs10993994 polymorphism on PC susceptibility, assuming an OR of 1.27.

However, potential limitations should be addressed in this meta-analysis. First, due to limited data, we could not perform further stratification analyses of other potential factors, such as age. Second, our results were based on unadjusted estimates for confounding factors, which might influence the final findings. Third, we could not assess potential gene-gene and gene-environment interactions. Fourth, the sample sizes of stratification analyses were limited in some ethnicities, such as Asians and African-Americans.

In summary, this meta-analysis indicates that MSMB gene rs10993994 polymorphism increased the risk of PC, especially among Caucasians. Further studies are necessary to validate whether this SNP is associated with RA susceptibility in other ethnic groups.

## MATERIALS AND METHODS

### Literature search and criteria of inclusion

We systematically searched the PubMed, and Embase to identify studies through June 15, 2016. The following search terms were used: “prostate cancer,” “PC,” “MSMB,” “Microseminoprotein-β,” “SNP” and “polymorphism”. No restrictions were placed on the literature search. Additional initially omitted studies were identified by hand screening. The inclusion criteria of studies were as following: (1) studies that evaluated the association between MSMB gene rs10993994 polymorphism and PC risk, (2) studied on human beings, (3) study provided sufficient data to calculate the odds ratios (ORs) and 95% confidence intervals (CIs), and *P* value, and (4) case-control study. Exclusion criteria were: (1) a duplication of previous publications; (2) a review, editorial or other non-original study; (3) studies without detailed genotype data, and (4) inclusion of subjects with other diseases that might influence the results.

### Data extraction and quality assessment

Data was extracted from all eligible studies by two reviewers. The extracted information from all eligible studies including: name of first author, publication year, ethnicity, source of controls, and genotype numbers of cases and controls. Two reviewers independently conducted the extraction of data and assessed the study quality according to the NOS [24]. All disagreements were resolved by discussion.

### Statistical analysis

The crude ORs and 95%CIs were used to assess the strength of associations between MSMB gene rs10993994 polymorphism and PC risk. Stratification analysis was carried out by ethnicity and SOC. When a Q test indicated *P* < 0.1 or I^2^ > 50% indicated heterogeneity across studies, a random-effect model was used. Otherwise, the fixed-effects model was applied [25]. Pooled ORs were calculated for allele model, dominant model, and recessive model. We performed leave-one-out sensitivity analysis to evaluate the stability of the overall results. We assessed the departure from the HWE in the controls using Pearson's χ2 test. Begger's and Egger's linear regression test were used to detect the potential publication bias [26]. The power of this meta-analysis was calculated at a significant value of 0.05 [27]. Meta-regression analysis of ethnicity and SOC was performed to seek the main sources of the heterogeneity. All statistical analyses were performed using the Stata 11.0 software (STATA Corporation, College Station, TX, USA).

## Abbreviations

PC: prostate cancer
MSMB: microseminoprotein-β
PAP: prostatic acid phosphatase
PSA: prostate-specific antigen
CI: confidence interval
OR: odds ratio
NOS: Newcastle-Ottawa Scale
HWE: Hardy–Weinberg equilibrium
SNP: single nucleotide polymorphism
GWAS: genome-wide association study
